# MicroRNA Expression in Myocardial Tissue and Plasma of Patients with End-Stage Heart Failure during LVAD Support: Comparison of Continuous and Pulsatile Devices

**DOI:** 10.1371/journal.pone.0136404

**Published:** 2015-10-02

**Authors:** Sjoukje I. Lok, Nicolaas de Jonge, Joyce van Kuik, Ankie J. P. van Geffen, Manon M. H. Huibers, Petra van der Weide, Erica Siera, Bjorn Winkens, Pieter A. Doevendans, Roel A. de Weger, Paula A. da Costa Martins

**Affiliations:** 1 Department of Cardiology, University Medical Center, Utrecht, the Netherlands; 2 Department of Pathology, University Medical Center, Utrecht, the Netherlands; 3 Department of Methodology and Statistics, Maastricht University, Maastricht, the Netherlands; 4 Department of Cardiology, CARIM School for Cardiovascular Diseases, Faculty of Health, Medicine and Life Sciences, Maastricht University, Maastricht, the Netherlands; IRCCS-Policlinico San Donato, ITALY

## Abstract

**Aim:**

Pulsatile flow left ventricular assist devices (pf-LVADs) are being replaced by continuous flow LVADs (cf-LVADs) in patients with end-stage heart failure (HF). MicroRNAs (miRs) play an important role in the onset and progression of HF. Our aim was to analyze cardiac miR expression patterns associated with each type of device, to analyze differences in the regulation of the induced cardiac changes.

**Methods and Results:**

Twenty-six miRs were selected (based on micro-array data and literature studies) and validated in myocardial tissue before and after pf- (n = 17) and cf-LVAD (n = 17) support. Of these, 5 miRs displayed a similar expression pattern among the devices (miR-129*, miR-146a, miR-155, miR-221, miR-222), whereas others only changed significantly during pf-LVAD (miR-let-7i, miR-21, miR-378, miR-378*) or cf-LVAD support (miR-137). In addition, 4 miRs were investigated in plasma of cf-LVAD supported patients (n = 18) and healthy controls (n = 10). Circulating miR-21 decreased at 1, 3, and 6 months after LVAD implantation. MiR-146a, miR-221 and miR-222 showed a fluctuating time pattern post-LVAD.

**Conclusion:**

Our data show a different miR expression pattern after LVAD support, suggesting that differentially expressed miRs are partially responsible for the cardiac morphological and functional changes observed after support. However, the miR expression patterns do not seem to significantly differ between pf- and cf-LVAD implying that most cardiac changes or clinical outcomes specific to each device do not relate to differences in miR expression levels.

## Introduction

Heart transplantation (HTx) is the ultimate therapy for patients with end-stage heart failure (HF), despite the discrepancy between the number of donor hearts available and the number of patients awaiting HTx. Left ventricular assist devices (LVADs) were introduced as a bridge to transplantation in order to reduce mortality and improve quality of life while waiting for HTx. LVADs provide volume and pressure unloading of the left ventricle, reversing the compensatory responses of the overloaded myocardium, resulting in partial “reverse remodeling” [1–4]. Clinical experience with LVAD support has shown that a subset of patients could be weaned from the device after restoration of basic cardiac function, so called bridge to recovery [1–6]. A lot of knowledge has emerged from studies on pulsatile flow LVADs (pf-LVADs), providing insights into the basic mechanisms and limitations of ventricular recovery [3,7]. Long-term LVAD support reduces cardiomyocyte size [8–10], decreases extracellular matrix volume [11,12]. Moreover, fibrillar collagen structure, β-adrenergic responsiveness and receptor density [13] and mitochondrial function improve [14]. These changes are paralleled by changes in the calcium handling properties of the cardiomyocytes, resulting in faster calcium entry via sarcolemmal calcium channels, higher sarcoplasmic reticulum calcium content and shorter action potential durations [15,16]. Currently, pf-LVADs have been replaced by continuous flow LVADs (cf-LVAD). Although many studies showed that cf-LVADs are as effective or even better in transplant rate and post-transplant outcomes [8,17,18], differences in left ventricular unloading between the devices may result in differences between “reverse remodeling” and the experience with pf-LVADs may no longer apply [19–21]. In addition, the lack of validated biomarkers for recovery aggravates uncertainties about ideal timing for potential LVAD explantation. A hallmark of HF mRNA signatures is that more transcripts are down regulated then up regulated, suggesting the importance of molecular mechanisms that suppress mRNA steady state levels. MicroRNAs (miRs) are small, non-coding RNAs that bind mRNAs at their 3’-untranslated regions, stimulating mRNA degradation or inhibiting protein translation [22]. MiRs are important hallmarks for recovery or deterioration and might be used as a therapeutic target in HF [23–25]. Therefore, delineating their role in posttranscriptional gene regulation offers new insight into the mechanisms how the heart adapts to mechanical support. Because in cf-LVAD, the left ventricle is constantly unloaded throughout the complete cardiac cycle and in pf-LVAD this occurs only at specific time points in the cycle, it is important to compare the physiological effects of such differences. In this context, our goal was to identify changes in miR expression during LVAD support and to investigate whether these expression patterns differ between pf-LVAD and cf-LVAD support, both at tissue and plasma levels. Assessing these differences can help identifying new regulatory players and mechanisms in reverse remodeling during mechanical support.

## Material and Methods

### Study Population

Only patients with end-stage non-ischemic dilated cardiomyopathy that were supported with a LVAD as bridge to transplantation (BTT) were included. Demographics are summarized in Tables 1 and 2. Twenty-six miRs were analyzed in myocardial tissue pre- and post-LVAD from individual pf-LVAD (n = 17) and cf-LVAD patients (n = 17) using a custom made quantitative polymerase chain reaction (Q-PCR)-array from Qiagen. Myocardial tissue was obtained outside the suture area of the inflow cannula.

**Table 1:** Demographics of the patients (n = 15) selected for microarray analysis (pre-screening).

	Tissue		Plasma
	pf-LVAD	cf-LVAD	cf-LVAD
n	5	5	5
Characteristic^a^			
Age, years	38 ± 7	49 ± 6	42 ± 6
Male, %	5 (100%)	4 (80%)	2 (40%)
HF duration (days)	261 (26–1023)	1747 (1531–3316)	1754 (723–1884)
Body Mass Index, kg/m2	22.7 ± 1.5	26.2 ± 1.86	24.1 ± 1.47
Diabetes mellitus, %	0 (0%)	0 (0%)	0 (0%)
Hypertension, %	0 (0%)	0 (0%)	0 (0%)
Hyperlipidemia, %	0 (0%)	0 (0%)	1 (20%)
CVA/TIA, %	1 (20%)	2 (40%)	2 (40%)
NYHA classification IV, %	5 (100%)	5 (100%)	5 (100%)
Etiology of non-ischemic DCM, %			
- Idiopathic	2 (40%)	3 (60%)	2 (40%)
- Familial^b^	1 (20%)	2 (40%)	3 (60%)
- Toxic (drugs)	1 (20%)	0 (0%)	0 (0%)
- Myocarditis	1 (20%)	0 (0%)	0 (0%)
CRTD/ICD before LVAD implant, %	2 (40%)	4 (80%)	3 (60%)
Type of LVAD			
- Heart Mate II	0 (0%)	5 (100%)	5 (100%)
- Heart Mate (X)VE	3 (60%)	0 (0%)	0 (0%)
- Thoratec	1 (20%)	0 (0%)	0 (0%)
- Novacor	1 (20%)	0 (0%)	0 (0%)
Days of LVAD support^c^	204 (180–301)	489 (238–897)	500 (260–1082)

HF, heart failure; CVA, cerebrovascular accident; TIA, transient ischemic attack; NYHA, New York Heart Association; DCM, dilated cardiomyopathy; CRTD, cardiac resynchronization therapy defibrillator; ICD, implantable cardioverter defibrillator.

^a^ Categorical data are presented as number (%), continuous data as mean±SEM or median (IQR), respectively.

^b^ Familial DCM is defined if the patient has one or more family members diagnosed with idiopathic DCM or has a first-degree relative with an unexplained sudden death under the age of 35 years.

^c^ Days of LVAD support are based on patients who already underwent HTx.

**Table 2:** Baseline demographics of the patients supported with a pulsatile flow LVAD (pf-LVAD) and continuous flow LVAD (cf-LVAD) (validation).

	Tissue		Plasma
	pf-LVAD	cf-LVAD	cf-LVAD
n	17	17	18
Characteristic^a^			
Age, years	45 ± 3	39 ± 3	45 ± 3
Male, %	14 (82%)	16 (94%)	14 (78%)
Body Mass Index, kg/m2	25.7 ± 1.8	22.8 ± 0.87	24.3 ± 1.3
HF duration (days)	1754 (750–2489)	398 (49–1344)	1872 (424–2343)
Smoking			
- No	9 (53%)	13 (76%)	11 (61%)
- Yes	3 (18%)	2 (12%)	2 (11%)
- Former	5 (29%)	2 (12%)	5 (28%)
Diabetes mellitus, %	1 (6%)	0 (0%)	2 (11%)
Hypertension, %	1 (6%)	0 (0%)	0 (0%)
Hyperlipidemia, %	2 (12%)	1 (6%)	2 (11%)
CVA/TIA before LVAD, %	6 (35%)	1 (6%)	5 (28%)
NYHA classification IV, %	17 (100%)	17 (100%)	18 (100%)
Etiology of non-ischemic DCM, %			
- Idiopathic	8 (47%)	10 (59%)	6 (33%)
- Familial^b^	6 (35%)	4 (24%)	7 (39%)
- Myocarditis	2 (12%)	1 (6%)	3 (17%)
- Toxic	0 (0%)	1 (6%)	1 (6%)
- Peripartum cardiomyopathy	0 (0%)	1 (6%)	0 (0%)
- Systemic disease	1 (6%)	0 (0%)	1 (6%)
CRTD/ICD before implantation, %	13 (76%)	8 (47%)	11 (61%)
Type of LVAD			
- HeartMate-II	0 (0%)	17 (100%)	18 (100%)
- HeartMate-(X)VE	12 (70%)	0 (0%)	0 (0%)
- Thoractec	3 (18%)	0 (0%)	0 (0%)
- Novacor	1 (6%)	0 (0%)	0 (0%)
- HeartMate-IP	1 (6%)	0 (0%)	0 (0%)
Days of LVAD support^c^	282 (197–512)	206 (190–317)	386 (227–510)

HF, heart failure; CVA, cerebrovascular accident; TIA, transient ischemic attack; NYHA, New York Heart Association; DCM, dilated cardiomyopathy; CRTD, cardiac resynchronization therapy defibrillator; ICD, implantable cardioverter defibrillator.

^a^Categorical data are presented as number (%), continuous data as mean±SEM or median (IQR), respectively.

^b^Familial DCM is defined if the patient has one or more family members diagnosed with idiopathic DCM or has a first-degree relative with an unexplained sudden death under the age of 35 years.

^c^Days of LVAD support are based on patients who already underwent HTx.

Furthermore, 4 miRs were studied in the plasma of 18 individual cf-LVAD supported patients prior to and 1, 3, and 6 months after implantation and prior to HTx (using Taqman Gene Expression Assays from Life Technologies). Control plasma was collected from 10 healthy individuals.

The experimental design of the study is depicted in Fig A in S1 File. The methods are described in detail in the supplemental data. Our study has been approved by the Institutional Review Board of the University Medical Center Utrecht, the Netherlands. All subjects included in this study gave written informed consent for the use of their tissues and plasma for research purposes.

### miR Selection Criteria

The selection of the investigated miRs was made based on the results from 2 microarray experiments performed on pf- and cf-LVAD myocardial tissue at Exiqon Services (Copenhagen, Denmark) according to the manufacturer’s protocol (supplemental data), combined with literature studies [24,26,27].

### Q-PCR Array Analysis of miR Expression in Tissue

The expression level of 26 miRs was investigated in 2 patient cohorts; pf-LVAD (n = 17) and cf-LVAD (n = 17) supported patients. RNA was isolated from 20 snap frozen 10μm sections of myocardial tissue (pre- and post-LVAD) using the miRNeasy Mini Kit (Qiagen Inc, Austin, USA) according to the manufacturer’s instructions. For cDNA synthesis and Q-PCR analysis, the miScript II RT kit and a custom made miScript PCR Array (based on the 26 selected miRs and 6 controls in a 96-wells format) were used (Qiagen Inc). In short, 250 ng of total RNA was converted into cDNA using the 5x miScript HiSpec buffer. Thermal cycling was done using the ViiA 7 Real-Time PCR system (Life Technologies, USA) and consisted of a 15 minute hot start at 95°C followed by 40 cycles of 94°C for 15 sec, 55°C for 30 sec and 70°C for 30 sec.

Four of the 6 controls on the custom made array were candidate stable references (miR-340, miR-664 were selected because of their stable expression in the previously performed Exiqon array. Also, SNORD68 and RNU6-2 were chosen because these small RNAs have been verified to have relatively stable expression levels across tissues (miScript Manual). The standard deviation (SD) of the Cq values of all samples was determined for the candidate reference genes (SD for miR-340 = 0.46, SD for miR-664 = 0.40, SD for RNU6-2 = 0.47, SD for SNORD68 = 0.48). Expression levels of miR-664 were used for data normalization, because the expression pattern of this miR was very stable among all samples tested. The other 2 controls were a reverse transcription control (miRTC) and a positive PCR control (PPC). The relative quantity (RQ) was calculated using the ΔΔCq method.

### Q-PCR Analysis of miR Expression in Plasma

From the custom array on tissue, 4 miRs were selected to further investigate their expression levels in plasma. RNA was isolated from 500-μl plasma at different time points (pre cf-LVAD support, 1, 3 and 6 months after cf-LVAD implantation, and pre-HTx) using the miRVana PARIS kit (part# AM1556, Life Technologies, USA) according to the manufacturer’s instructions. cDNA synthesis for miRs was performed using the TaqMan MicroRNA Reverse Transcription Kit Life Technologies, USA). Q-PCR was performed using TaqMan MicroRNA assays (Life Technologies, USA). PCR reactions were performed on the ViiA 7Real-Time PCR system (Life Technologies, USA). Thermal cycling conditions consisted of a denaturation step at 95°C for 10 min, followed by 40 cycles of 95°C for 15 sec and 60°C for 1 min. Cq values above 35 were defined negative. A stable miR (miR-148b) from a previously performed Q-PCR array (Exiqon) on plasma was used as reference and the relative quantity (RQ) was calculated using the ΔΔCq method.

### Statistical Analysis

Categorical and continuous data are presented by number (%) and by mean (SD) or median (interquartile range IQR, i.e. 25th- 75th percentile), where appropriate. Independent-samples t-tests were used for comparing miR expression arrays (Exiqon). Due to the high number of miRs being tested in parallel, micro-arrays are prone to give false positive results. The false positive rate is controlled by multiple testing corrections that adjust p-values derived from multiple statistical tests. Therefore, p-values were calculated with and without the Benjamin and Hochberg multiple testing adjustment method [28]. For miR Q-PCR array (Qiagen) data, the groups were compared using paired-samples t-test and Wilcoxon signed rank test, where appropriate. For miR expression profiling in plasma, the longitudinal detection pattern of circulating miRs (from pre-LVAD until pre-Htx) was evaluated using the mixed model analysis with a random intercept to account for the correlation between repeated measurements within the same person. The data from this model are presented by estimated mean ± standard error. The miR expression pre-LVAD was compared with healthy controls using Mann-Whitney U test. Furthermore, also the changes in miR-expression between individual patients pre/post pf-LVAD and pre/post cf-LVAD were calculated by using Mann-Whitney U test. P-values ≤ 0.05 were considered statistically significant. Mixed model analysis was applied using SPSS, version 20 (SPSS, Inc., Chicago, Illinois, USA). All other analyses were performed using GraphPad Prism version 5.0 (GraphPad Software Inc., La Jolla, CA, USA).

## Results

### Patient Demographics

Patients supported with a pf-LVAD (n = 17) had a mean age of 45 years, 82% (n = 14) were male, with a median HF duration of 1754 days (IQR 750–2489) and LVAD duration of 282 (IQR 197–512) days (**Tables 1 and 2**). The majority of the patients (70%) was supported with a HeartMate (X)VE. The cf-LVAD supported group (n = 17) consisted of patients with a mean age of 39 years and 94% (n = 16) were male. The median duration of HF was 398 days (IQR 49–1344) and the LVAD duration was 206 (IQR 190–317) days. In the cf-LVAD group, all patients were supported with a HeartMate II device (**Table 2**).

**Fig 1:**
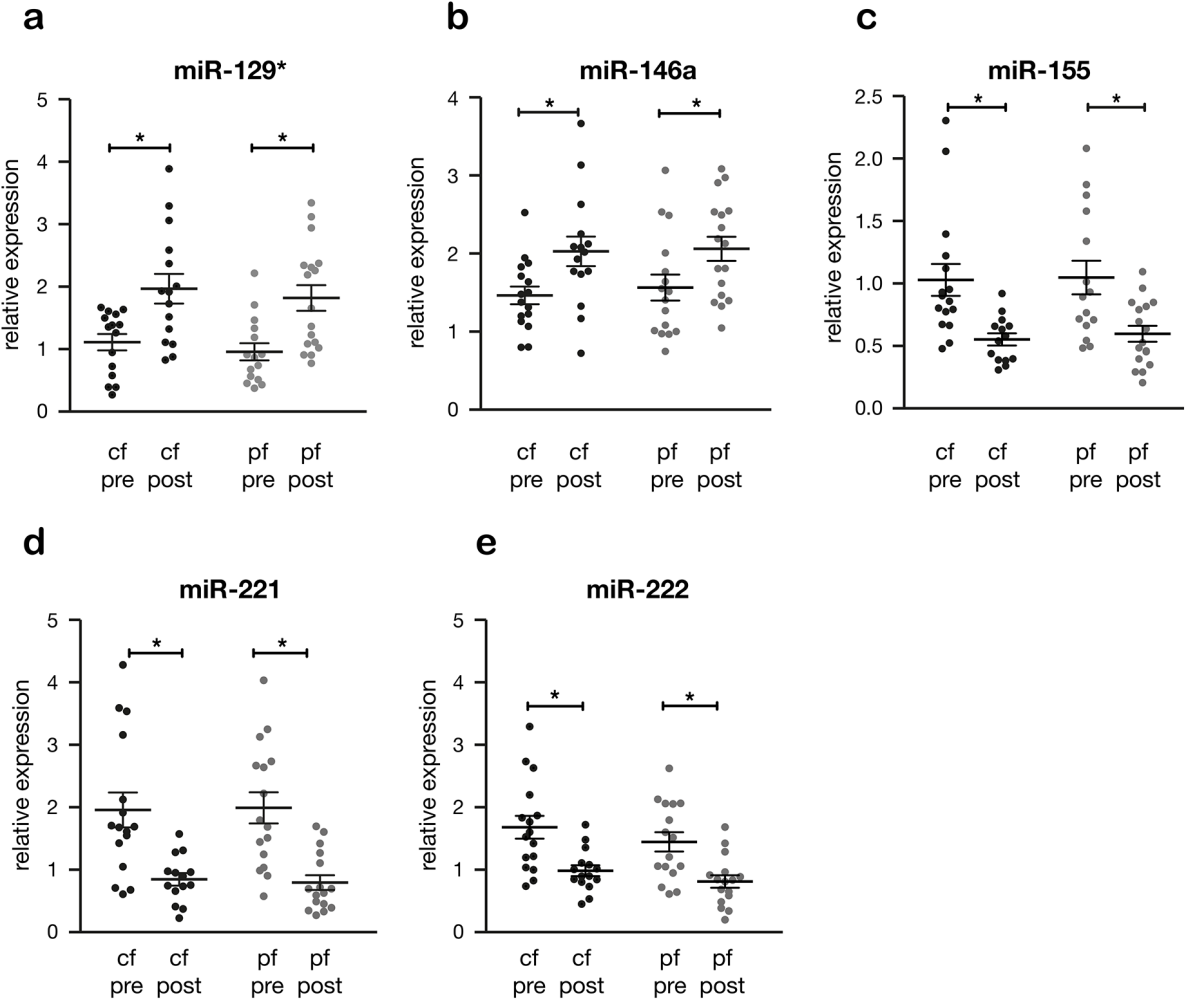
Similar changes in miR expression in pulsatile flow (pf-LVAD) and continuous flow LVAD (cf-LVAD). The alteration in miR-expression in myocardial tissue of both pf-LVAD (n = 17) and cf-LVAD (n = 17) patients. miR-129* (*a*) and miR-146a (*b*) are up-regulated in post-LVAD in comparison to pre-LVAD, whereas miR- 155 (*c*), miR-221 (*d*) and miR-222 (*e*) are down-regulated in both devices. The asterisk (*) represents p<0.05.

Out of the 26 selected miRs (Table A in S1 File), 5 miRs showed a similar pattern during pf-LVAD and cf-LVAD support (tables A-C in **S2 File and S3 File**). MiR-129* and miR-146a were down-regulated in pre-LVAD supported patients and increased during mechanical support (**Fig 1A and 1B**). In contrast, miR-155, miR-221 and miR-222 were up-regulated in pre-LVAD and decreased upon implantation (**Fig 1C–1E**).

**Fig 2:**
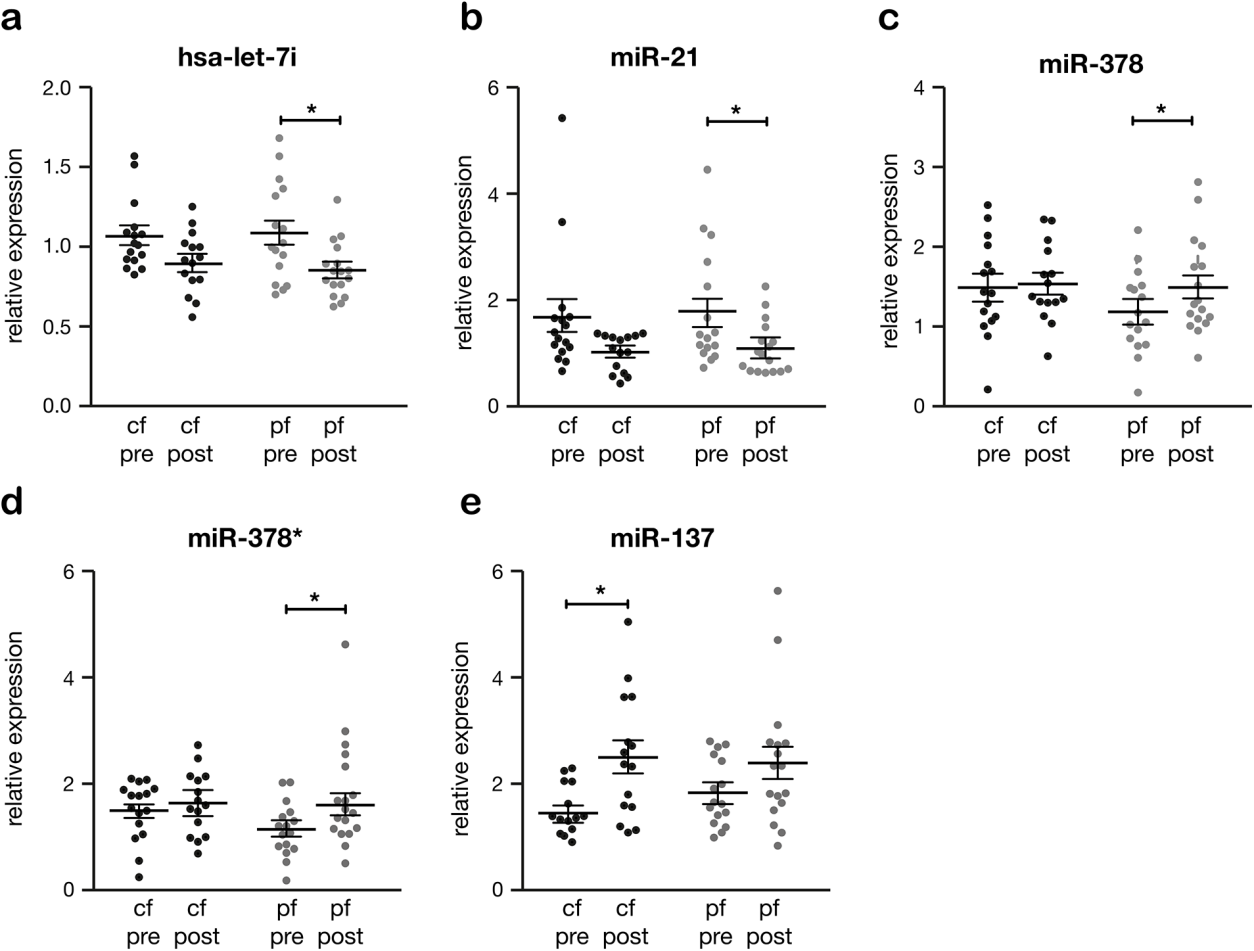
Device specific changes in miR expression. Selection of myocardial miRs that changed only in pulsatile flow (pf-LVAD) or continuous flow LVAD (cf- LVAD), respectively. During pf-LVAD support, miR-let-7i (*a*), miR-21 (*b*), miR-378 (*c*) and miR-378* (*d*) changed significantly post LVAD, whereas in cf-LVAD these miRs did not differ significantly. In cf-LVAD, only miR-137 (*e*) changed significantly post-LVAD. The asterisk (*) represents p<0.05.

Several miRs showed a significant change in either pf-LVAD or cf-LVAD support. Four miRs changed in expression after pf-LVAD (miR-let-7i, miR-21, miR-378 and miR-378*, **Fig 2A–2D**), whereas the expression of miR-137 only changed significantly during cf-LVAD support (**Fig 2E**). Of note, let-7i and miR-21 changed non-significantly in cf-LVAD tissue, but showed the same trend as in pf-LVAD. In fact, statistical analysis to investigate whether the difference in expression of these four microRNAs really differ between pf-LVAD and cf-LVAD support revealed that the effects were not significant when comparing the change pre- and post-LVAD within individuals (Fig B in S1 File). Interestingly, several miRs that have previously been reported to be differentially expressed between pre- and post pf-LVAD [7], did not significantly change in cf-LVAD. This was the case for miR-133a, miR-133b, miR-1, miR-151, miR-7a and miR-378 (Fig C in S1 File).

**Fig 3:**
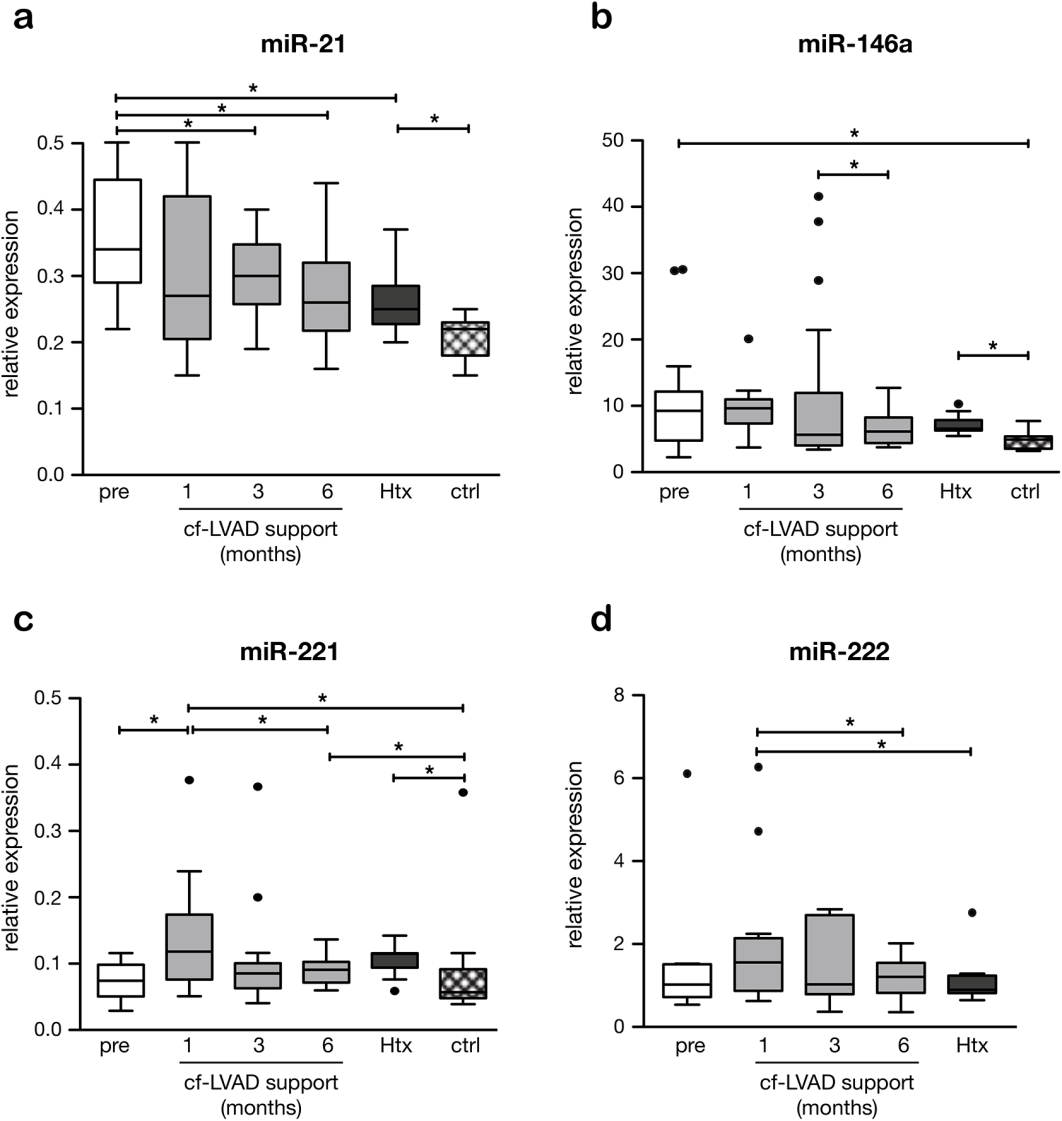
Changes in plasma miR expression after continuous flow LVAD (cf-LVAD) implantation. Expression of circulating miR-21 (*a*), miR-146a (*b*), miR-221 (*c*) en miR-222 (*d*) during cf-LVAD support prior to and 1,3, and 6 months after implantation and before HTx (n = 18 patients) and controls (n = 10). In miR-222, no reliable duplicates on controls could be measured (*d*). The asterisk (*) represents p<0.05.

4 miRs that changed significantly in cf-LVAD supported patients were selected for further validation in plasma by Q-PCR: miR-21, miR-146a, miR-221 and miR-222. Prior to cf-LVAD implantation, levels of miR-21 were doubled compared to controls, decreased significantly during support, but did not normalize (**Fig 3A**). Levels of miR-146a were also increased in pre-LVAD compared to controls, but revealed a fluctuating expression pattern during support with a tendency to decrease (**Fig 3B**). The expression patterns for miR-221 and miR-222 were alike, with both miRs up-regulated after 1 month followed by down-regulation (**Fig 3C and 3D**). While for miR-21 the time response in expression levels was homogeneous between the different patients, for the other three microRNAs, the expression levels determined in a few patients (outliers, non-responders) are very discrepant from the rest of the group (**Fig 3A–3D**).

## Discussion

In the present study, we compare the miR expression patterns in pulsatile flow (pf-) versus continuous flow (cf-) LVAD-supported myocardium.

The use of cf-LVADs has been increasing over the last years and this is mostly related to its many advantages over pf-LVADs: smaller size, reducing surgical trauma, higher mechanical reliability, limited-blood containing surfaces, better energy efficiency and decreased risk of device-related infections [8,10]. Nevertheless, because cf-LVADs generate less pulsatile hemodynamics, there are still some reservations regarding the effect of long-term continuous blood flow mainly regarding cardiovascular remodeling, perfusion of end organs and long-term patient outcome. Accordingly, it has been suggested that bridge to recovery is more likely in pf-LVAD support than in cf-LVAD support [11]. Whereas, most studies, so far, have compared the myocardial contractile profiles and cellular/hemodynamic responses of the failing myocardium in pf-LVAD versus cf-LVADs, the exact biochemical and molecular changes related to each type of device are still unclear. This study applies a miR expression profiling strategy to evaluate the molecular regulatory role of miRs during long-term LVAD support and to provide more insight in the molecular mechanisms that are affected by each type of device. miRs are established main players in the pathological cardiac remodeling process leading to HF [23,29–31]. LVAD support is able to partially reverse structural and mechanistic changes and this may be related to changes in miR expression patterns. To date, few studies have addressed changes in miRs during LVAD support [7,26,27,32–34]. Ramani *et al* examined the expression levels of 376 miRs in myocardial tissue of 28 patients to determine whether there was an association between the expression levels at the time of LVAD implantation and the ability of the heart to recover during support [33]. In addition, 7 non-failing hearts were investigated. Fourteen of the patients underwent removal of their LVAD. The level of miR-15b, miR-23a, miR-26a and miR-195 were significantly decreased in the left ventricle of the recovery hearts when compared to LVAD dependent hearts. Remarkably, LVAD support did not alter the expression of any of these miRs. Moreover, the expression levels of miR-23a and miR-195 in the recovery group were similar to those of non-failing hearts. An editorial comment of Mann and Burkhoff suggests therefore, that degree of recovery may relate more to the severity and nature of the underlying HF at the time of LVAD implantation, rather than to the effect of mechanical unloading [35]. Matkovich *et al* performed a parallel miR and mRNA micro-array profiling on myocardial tissue from non-failing hearts, failing hearts, and failing hearts supported with a LVAD [32]. They showed that 28 miRs were 2-fold increased in failing hearts, and there was near complete normalization of the miR signature in the LVAD supported hearts. By contrast, 444 mRNAs were altered by 1.3 fold in failing hearts, and only 29 of these normalized by 25% in the LVAD supported hearts. This suggests that miRs may be more sensitive than mRNAs to functional changes related to end-stage HF. In fact, previous work from our group [7] focusing on the differential expression of four specific miRs known to be expressed in the heart and to have functional roles in progression of HF (miR-1, miR-133a, miR-133b and miR-208), showed that expression of these miRs is restored during pf-LVAD support. However, this was only observed in ischemic heart disease patients and not in patients with dilated cardiomyopathy. Similarly, in the present study, we showed that expression of these miRs did not change significantly in cf-LVAD supported myocardial tissue of patients with dilated cardiomyopathy (fig **C in S1 File**).

The present study shows that there is a specific set of miRs with the same expression pattern changes after cf- and pf-LVAD support, with some of them being restored to expression levels of controls after LVAD support. Curiously, when examining the temporal expression pattern of circulating miR-21 after implantation and just before HTx, it revealed a consistent decreasing pattern until control levels were reached. miR-21 is very abundant in the cardiovascular system and several studies have revealed that its expression is deregulated in heart and vasculature under cardiovascular disease conditions, such as proliferative vascular disease, cardiac hypertrophy, HF, and ischemic heart disease [36–38]. There are, however, some miRs that were further depleted or induced after support and therefore, have an expression behavior very different from healthy hearts. miR-221 and miR-222, are examples of miRs that are repressed in HF and this repression is even more accentuated after support. This kind of response may reflect the role of these miRs in regulating vascular inflammation and therefore, their function as mediators of endothelial cell pathophysiology [39,40] may provide clues as to why cardiac function is not completely normalized in most LVAD patients. The lack of donor hearts as control myocardial tissue constitutes a limitation. Hence, it is difficult to draw significant conclusions comparing non-failing versus LVAD supported hearts.

At first, our data seems to confirm the hypothesis that different type of support devices result in different miR expression profiles. From all the differentially expressed miRs in LVAD-supported myocardium, only 30% are common to both types of devices, suggesting that all others are specific for cf- or pf-LVAD. In agreement, as miR-137 seems to be a cf-LVAD-specific miR, miRs such as miR- let7i, -21, -378 and -378* show a more pronounced change in pf-LVAD as compared to cf-LVAD. Remarkably, all these miRs are not only implicated in vascular inflammation but also in cell growth and vascularization in several types of cancer [41,42]. miR-378 and miR-378* have also been implicated in cardiac pathological remodeling as being negative regulators of cardiac hypertrophic growth [43,44]. In agreement, our results show a significant increase in miR-378 and miR-378* in post pf-LVAD, indicating that increased expression levels of these miRs contribute to better cardiac function and therefore positive cardiac remodeling. Furthermore, we have very recently shown that miR-137 is implicated in reverse remodeling during cf-LVAD support by directly regulating alpha-1-antichymotrypsin, a potential new biomarker of HF [25].

These differences between miR expression profiles related to cf- and pf-LVAD could explain the different patient outcome observed after myocardial support by each type of device. In fact, previous profiling studies have detected that mRNA signatures do not always reflect the improvements delivered by biomechanical support such as that provided by LVADs [45,46]. From a total of 3088 mRNA transcripts exhibiting abnormal abundance in HF, only 11% of these genes exhibit partial recovery and only 5% showed true normalization [45]. These numbers may reflect the fact that not all secondary effects of LVAD support are beneficial. In a similar way, also the specific miR expression profiles induced by either pulsatile or continuous flow will affect the clinical outcome of those patients either by restoring protein levels (in some cases just to a certain extent) or even accentuate the unbalance between pre and post-LVAD.

However, when comparing individual pre- and–post LVAD differences for some of the miRs that were differentially expressed after LVAD, we could observe that the differences in expression change between the two devices were not significant. These results question the relevance of the changes observed within each group. Although future studies including larger groups of patients and controls are necessary to clarify some remaining questions, altogether, our data strongly suggests that similar signaling molecules are implied in the reverse remodeling associated to pulsatile- and continuous flow LVAD support, also indicating that further studies are necessary to understand the molecular and cellular basis of the device-specific clinical outcomes.

## Supporting Information

S1 File Experimental design of the study (Fig A); Differences between individual changes in myocardial tissue (pre- and–post) in pf-LVAD versus cf-LVAD support. (Fig B); Selected miRs in myocardial tissue (pre- and post) in pf-LVAD versus cf-LVAD support (Fig C); Selected miRs (table A).
(DOC)

S2 File Microarray analysis of miR expression in cardiac tissue of pf-LVAD patients; pre-LVAD versus post-LVAD (table A); pre-LVAD versus control (table B) and post-LVAD versus control (table C). (XLSX)

S3 File Microarray analysis of miR expression in cardiac tissue of cf-LVAD patients; pre-LVAD versus post-LVAD (table A); pre-LVAD versus control (table B) and post-LVAD versus control (table C). (XLSX)

S4 File Raw data miR expression in cardiac tissue of pf-LVAD patients; pre-LVAD versus post-LVAD (table A); pre-LVAD versus control (table B); post-LVAD versus control (table C). (XLSX)

S5 File Raw data miR expression in cardiac tissue of cf-LVAD patients; pre-LVAD versus post-LVAD (table A); pre-LVAD versus control (table B) and post-LVAD versus control (table C). (XLSX)
