# Supplementary material for: MicroRNA Expression in Myocardial Tissue and Plasma of Patients with End-Stage Heart Failure during LVAD Support: Comparison of Continuous and Pulsatile Devices
Source: PLoS One. 2015 Oct 2;10(10):e0136404. doi: 10.1371/journal.pone.0136404 (PMC4592005; doi:10.1371/journal.pone.0136404)
Supplement: S1 File — (Fig B); Selected miRs in myocardial tissue (pre- and post) in pf-LVAD versus cf-LVAD support (Fig C); Selected miRs (table A). (DOC) [file pone.0136404.s001.doc]

**Supplemental data**

**Methods and materials**

*Tissue and plasma arrays (Pre-screening)*

*For tissue*: Myocardial tissue was collected pre- and post pf/cf-LVAD implantation. Total RNA was isolated from 20 sections of 10-µm snap-frozen tissue using miRNeasy Mini Kit according to the manufacturer’s instructions (Qiagen Inc, Austin, USA). The RNA quality was verified by an Agilent 2100 Bioanalyzer profile. 500 ng total RNA from both samples and reference were labeled with a Hy3 and HY5 fluorescent label, respectively, using the miRCURY LNA microRNA array Hi-Power Labeling Kit, Hy3/HY. The Hy3-labeled samples and the HY5-labeled reference RNA sample were mixed pair-wise and hybridized to the miRCURY LNA microRNA Array (6^th^ Gen, consisting of 3100 probe sets). The quantified signals were background corrected (Normexp with offset value 10) and normalized using the global Lowess (LOcally WEighted Scatterplot Smoothing) regression algorithm. Data quality assessment showed that the labeling was successful for all capture probes since the control spike-in oligo nucleotides produced signals in the expected range. Samples that showed lowered correlation of the spike-on-controls compared to the remaining samples were, therefore, excluded from further analysis.

*For plasma*: Plasma from patients was collected prior to cf-LVAD implantation and 6 months thereafter. Total RNA was isolated from 200µl plasma per sample using miRNeasy Mini Kit (Qiagen Inc). Each RNA sample was reverse transcribed into cDNA and run on the miRCURY LNA Universal RT miR PCR Human panel I and II, which contain together, 742 miR assays. Each PCR panel contained a PCR control (three replicates of an inter-plate calibrator) and 3 primer sets for reference genes. In addition, one enzyme control (reverse transcriptase replaced by water) was included as negative control. The negative controls were used to set the threshold for sample detection, to which a value of 42 was assigned. The criterion for including an assay in the further analysis is that the quantification cycle (Cq) must be 5 cycles lower than the negative control.

An additional step in the real time PCR analysis, by generating a melting curve for each reaction, was performed to evaluate the specificity of the assays. Any assays that showed multiple peaks have been excluded from the data set. The amplification curves were analyzed using the Roche LC software, both for determination of Cq and for melting curve analyses. PCR efficiency was also assessed by analysis of the PCR amplification curve using algorithms similar to the LinReg software. The average efficiencies ranged between 1.8 and 2.1. Individual reactions that gave an efficiency<1.6 were excluded from the dataset. The melting temperature was furthermore checked to be within the specifications of the individual assays. For normalization of the data, the average of the assays detected in all samples has been applied. These were 152 assays. By using the formula *normalized Cq = Average Cq (n=152) – assay Cq* to calculate the normalized Cq values, lower values will indicate that a specific miR is more abundant in that specific sample.

RNA was isolated from 20 snap frozen 10µm sections of myocardial tissue (pre- and post-LVAD) using the miRNeasy Mini Kit (Qiagen Inc, Austin, USA) according to the manufacturer. For cDNA synthesis and Q-PCR analysis, the miScript II RT kit and a custom made miScript PCR Array (based on the 26 selected miRs and 6 controls and in a 96-wells format) were used (Qiagen Inc). In short, 250 ng of total RNA was converted into cDNA using the 5x miScript HiSpec buffer. Thermal cycling was done using the ViiA 7 Real-Time PCR system (Life Technologies, USA) and consisted of a 15 minute hot start at 95°C followed by 40 cycles of 94°C for 15 sec, 55°C for 30 sec and 70°C for 30 sec.

Four of the 6 controls on the custom made array were candidate stable references (miR-340, miR-664 were selected because of their stable expression in the previously performed Exiqon array. Also, SNORD68 and RNU6-2 were chosen because these small RNAs have been verified to have relatively stable expression levels across tissues (miScript Manual). The standard deviation (SD) of the Cq values of all samples was determined for the candidate reference genes (SD for miR-340 =0.46, SD for miR-664 = 0.40, SD for RNU6-2 = 0.47, SD for SNORD68 = 0.48). Expression levels of miR-664 were used for data normalization, because the expression pattern of this miR was very stable among all samples tested. The other 2 controls were a reverse transcription control (miRTC) and a positive PCR control (PPC). The relative quantity (RQ) was calculated using the ΔΔCq method.

*Q-PCR analysis of miR expression in tissue*

For cDNA synthesis and Q-PCR analysis, the miScript II RT kit and a custom made miScript PCR Array (based on the 26 selected miRs and 6 controls and in a 96-wells format) were used (Qiagen Inc). In short, 250 ng of total RNA was converted into cDNA using the 5x miScript HiSpec buffer. Thermal cycling was done using the ViiA 7 Real-Time PCR system (Life Technologies, USA) and consisted of a 15 minute hot start at 95°C followed by 40 cycles of 94°C for 15 sec, 55°C for 30 sec and 70°C for 30 sec.

Four of the 6 controls on the custom made array were candidate stable references (miR-340, miR-664 were selected because of their stable expression in the previously performed Exiqon array. Also, SNORD68 and RNU6-2 were chosen because these small RNAs have been verified to have relatively stable expression levels across tissues (miScript Manual). The standard deviation (SD) of the Cq values of all samples was determined for the candidate reference genes (SD for miR-340 =0.46, SD for miR-664 = 0.40, SD for RNU6-2 = 0.47, SD for SNORD68 = 0.48). Expression levels of miR-664 were used for data normalization, because the expression pattern of this miR was very stable among all samples tested. The other 2 controls were a reverse transcription control (miRTC) and a positive PCR control (PPC). The relative quantity (RQ) was calculated using the ΔΔCq method.

*Q-PCR analysis of miR expression in plasma*

cDNA synthesis for miRs was performed using the TaqMan MicroRNA Reverse Transcription Kit Life Technologies, USA). Q-PCR was performed using TaqMan MicroRNA assays (Life Technologies, USA). PCR reactions were performed on the ViiA 7Real-Time PCR system (Life Technologies, USA). Thermal cycling conditions consisted of a denaturation step at 95 °C for 10 min, followed by 40 cycles of 95 °C for 15 sec and 60 °C for 1 min. Cq values above 35 were defined negative. A stable miR from the previously performed Exiqon array on plasma (miR-148b) was used as reference and the relative quantity (RQ) was calculated using the ΔΔCq method.

**Supplemental figures and tables**

**S1 file.**

Figure A. Experimental design of the study

Figure B. Differences between individual changes in myocardial tissue (pre- and –post) in pf-LVAD versus cf-LVAD support.


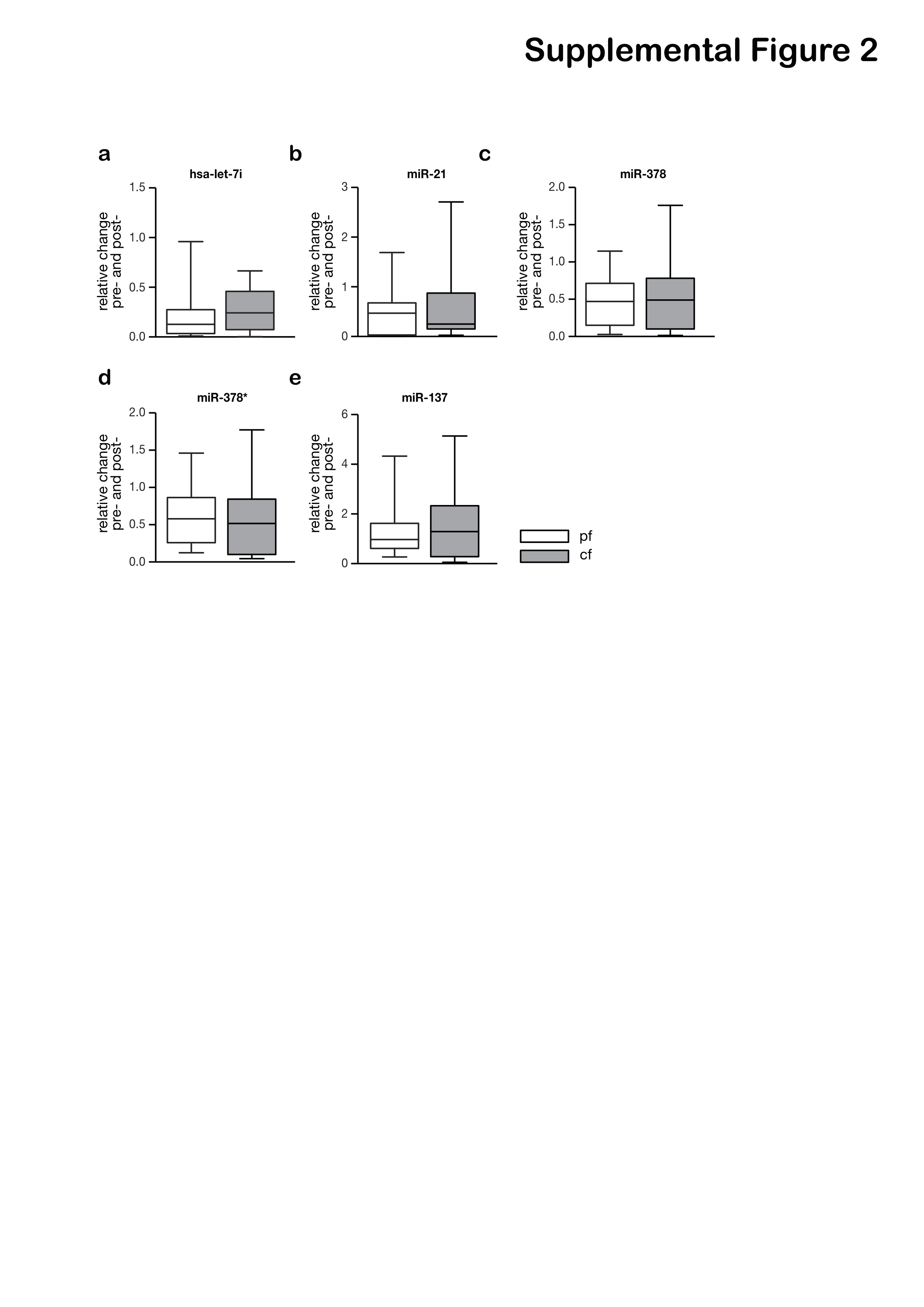


Legend: Comparison of differences between individual changes pre- and –post in pf-LVAD and cf-LVAD showing no significant differences between devices.

Figure C. Selected miRs in myocardial tissue (pre- and post) in cf-LVAD support

Legend: These selected miRs have previously been investigated in pf-LVAD supported patients and changed significantly. Whereas this figure represents no significant change in miR expression during cf-LVAD support.

| Table A**.** Selection of miRs. | |
| --- | --- |
|  |  |
| **Annotation** | **Selection** |
| hsa-miR-208a | literature (1-4) |
| hsa-let-7i | literature (5,6) |
| hsa-miR-137 | literature (7) |
| hsa-miR-23a* | literature (8-10) |
| hsa-miR-199b-5p | literature (11,12) |
| hsa-miR-320d | literature (13,14) |
| hsa-miR-133b | literature (1,15-18) |
| hsa-miR-21 | literature (19-23) |
| hsa-miR-199a-5p | micro-array |
| hsa-miR-29b-1* | micro-array |
| hsa-miR-17* | micro-array |
| hsa-miR-92a | micro-array |
| hsa-miR-25 | micro-array |
| hsa-miR-146a | micro-array |
| hsa-miR-221 | micro-array |
| hsa-miR-23a | micro-array |
| hsa-miR-378 | micro-array |
| hsa-miR-1 | micro-array |
| hsa-miR-155 | micro-array |
| hsa-miR-22 | micro-array |
| hsa-miR-378* | micro-array |
| hsa-miR-129* | micro-array |
| hsa-miR-142-5p | micro-array |
| hsa-miR-136 | micro-array |
| hsa-miR-222 | micro-array |

Legend: List of miRs that were evaluated by real time PCR in individual patients before and after pf/cf-LVAD support.

**Supplemental references**

1. Widera C, Gupta SK, Lorenzen JM, Bang C, Bauersachs J, Bethmann K, Kempf T, Wollert KC, Thum T. Diagnostic and prognostic impact of six circulating microRNAs in acute coronary syndrome. *J Mol Cell Cardiol*. 2011; 51:872–875.

2. Montgomery RL, Hullinger TG, Semus HM, Dickinson BA, Seto AG, Lynch JM, Stack C, Latimer PA, Olson EN, van Rooij E. Therapeutic inhibition of miR-208a improves cardiac function and survival during heart failure. *Circulation*. 2011; 124:1537–1547.

3. Oliveira-Carvalho V, Carvalho VO, Bocchi EA. The emerging role of miR-208a in the heart. *DNA Cell Biol.* 2013; 32:8–12.

4. Callis TE, Pandya K, Seok HY, Tang R-H, Tatsuguchi M, Huang Z-P, Chen J-F, Deng Z, Gunn B, Shumate J, Willis MS, Selzman CH, Wang D-Z. MicroRNA-208a is a regulator of cardiac hypertrophy and conduction in mice. *J. Clin. Invest.* 2009; 119:2772–2786.

5. Satoh M, Minami Y, Takahashi Y, Tabuchi T, Nakamura M. A cellular microRNA, let-7i, is a novel biomarker for clinical outcome in patients with dilated cardiomyopathy. *J Card Fail*. 2011; 17:923–929.

6. Satoh M, Tabuchi T, Minami Y, Takahashi Y, Itoh T, Nakamura M. Expression of let-7i is associated with Toll-like receptor 4 signal in coronary artery disease: effect of statins on let-7i and Toll-like receptor 4 signal. *Immunobiology*. 2012; 217:533–539.

7. Lok SI, van Mil A, Bovenschen N, van der Weide P, van Kuik J, Van Wichen D, Peeters T, Siera E, Winkens B, Sluijter JPG, Doevendans PA, da Costa Martins PA, de Jonge N, de Weger RA. Post-transcriptional Regulation of α-1-Antichymotrypsin by MicroRNA-137 in Chronic Heart Failure and Mechanical Support. *Circ Heart Fail*. 2013; 6:853–861.

8. Wang J, Xu R, Lin F, Zhang S, Zhang G, Hu S, Zheng Z. MicroRNA: novel regulators involved in the remodeling and reverse remodeling of the heart. *Cardiology*. 2009; 113:81–88.

9. Lin Z, Murtaza I, Wang K, Jiao J, Gao J, Li P-F. miR-23a functions downstream of NFATc3 to regulate cardiac hypertrophy. *Proc. Natl. Acad. Sci. U.S.A.* 2009; 106:12103–12108.

10. Wang K, Lin Z-Q, Long B, Li J-H, Zhou J, Li P-F. Cardiac hypertrophy is positively regulated by MicroRNA miR-23a. *J Biol Chem*. 2012; 287:589–599.

11. Greco S, Fasanaro P, Castelvecchio S, D'Alessandra Y, Arcelli D, Di Donato M, Malavazos A, Capogrossi MC, Menicanti L, Martelli F. MicroRNA dysregulation in diabetic ischemic heart failure patients. *Diabetes*. 2012; 61:1633–1641.

12. da Costa Martins PA, Salic K, Gladka MM, Armand A-S, Leptidis S, Azzouzi el H, Hansen A, Coenen-de Roo CJ, Bierhuizen MF, van der Nagel R, van Kuik J, de Weger R, de Bruin A, Condorelli G, Arbones ML, Eschenhagen T, De Windt LJ. MicroRNA-199b targets the nuclear kinase Dyrk1a in an auto-amplification loop promoting calcineurin/NFAT signalling. *Nat. Cell Biol.* 2010; 12:1220–1227.

13. Ren X-P, Wu J, Wang X, Sartor MA, Qian J, Jones K, Nicolaou P, Pritchard TJ, Fan G-C. MicroRNA-320 is involved in the regulation of cardiac ischemia/reperfusion injury by targeting heat-shock protein 20. *Circulation*. 2009; 119:2357–2366.

14. Wang XH, Qian RZ, Zhang W, Chen SF, Jin HM, Hu RM. MicroRNA-320 expression in myocardial microvascular endothelial cells and its relationship with insulin-like growth factor-1 in type 2 diabetic rats. *Clin. Exp. Pharmacol. Physiol.* 2009; 36:181–188.

15. Bostjancic E, Zidar N, Stajer D, Glavac D. MicroRNAs miR-1, miR-133a, miR-133b and miR-208 are dysregulated in human myocardial infarction. *Cardiology*. 2010; 115:163–169.

16. Schipper MEI, van Kuik J, de Jonge N, Dullens HFJ, de Weger RA. Changes in regulatory microRNA expression in myocardium of heart failure patients on left ventricular assist device support. *J Heart Lung Transplant*. 2008; 27:1282–1285.

17. Xiao L, Xiao J, Luo X, Lin H, Wang Z, Nattel S. Feedback remodeling of cardiac potassium current expression: a novel potential mechanism for control of repolarization reserve. *Circulation*. 2008; 118:983–992.

18. Sucharov C, Bristow MR, Port JD. miRNA expression in the failing human heart: functional correlates. *J Mol Cell Cardiol*. 2008; 45:185–192.

19. Yang K-C, Ku Y-C, Lovett M, Nerbonne JM. Combined deep microRNA and mRNA sequencing identifies protective transcriptomal signature of enhanced PI3Kα signaling in cardiac hypertrophy. *J Mol Cell Cardiol*. 2012; 53:101–112.

20. Hu S, Huang M, Nguyen PK, Gong Y, Li Z, Jia F, Lan F, Liu J, Nag D, Robbins RC, Wu JC. Novel microRNA prosurvival cocktail for improving engraftment and function of cardiac progenitor cell transplantation. *Circulation*. 2011; 124:S27–34.

21. Patrick DM, Montgomery RL, Qi X, Obad S, Kauppinen S, Hill JA, van Rooij E, Olson EN. Stress-dependent cardiac remodeling occurs in the absence of microRNA-21 in mice. *J. Clin. Invest.* 2010; 120:3912–3916.

22. da Costa Martins PA, De Windt LJ. miR-21: a miRaculous Socratic paradox. *Cardiovasc. Res.* 2010; 87:397–400.

23. Thum T, Gross C, Fiedler J, Fischer T, Kissler S, Bussen M, Galuppo P, Just S, Rottbauer W, Frantz S, Castoldi M, Soutschek J, Koteliansky V, Rosenwald A, Basson MA, Licht JD, Pena JTR, Rouhanifard SH, Muckenthaler MU, Tuschl T, Martin GR, Bauersachs J, Engelhardt S. MicroRNA-21 contributes to myocardial disease by stimulating MAP kinase signalling in fibroblasts. *Nature*. 2008; 456:980–984.
